# μ-(2,6-Bis{[3-(di­methyl­amino)­prop­yl]imino­meth­yl}-4-methyl­phenolato)-μ-hydroxido-bis­[(thio­cyanato-κ*N*)copper(II)]

**DOI:** 10.1107/S1600536813024768

**Published:** 2013-09-12

**Authors:** M. G. Meera, P. Kamatchi Selvaraj, B. Viswanathan, V. Ramkumar

**Affiliations:** a Research and Development Centre, Bharathiar University, Tamil Nadu, India; b Department of Chemistry, Government Arts College, Nandanam, Chennai 600035, Tamil Nadu, India; c National Centre for Catalysis Research, IIT Madras, Chennai 600 036, TamilNadu, India; d Department of Chemistry, IIT Madras, Chennai 600 036, TamilNadu, India

## Abstract

In the title compound, [Cu_2_(C_19_H_31_N_4_O)(OH)(NCS)_2_], the mol­ecular structure of the dinuclear complex reveals two penta­coordinated Cu^II^ ions, which are bridged by the phenolate O atom of the ligand and by an exogenous hydroxide ion. The bridging atoms occupy equatorial positions in the coordination sphere of the metal atoms and complete the equatorial coordination planes with two ligand N atoms, the apical positions being occupied by thio­cyanate N atoms. The crystal structure also features π–π stacking inter­actions involving the benzene rings with a centroid–centroid distance of 3.764 (4)Å. The crystal studied was a non-merohedral twin, with a refined BASF value of 0.203 (2)

## Related literature

For related structures, see: Matsufuji *et al.* (2005 ▶); Amase *et al.* (2005 ▶); Erxleben & Hermann (2000 ▶); Higuchi *et al.* (1995 ▶); Koga *et al.* (1998 ▶); Knight *et al.* (2008 ▶). For applications and properties of binuclear copper (II) complexes, see: Adams *et al.* (2000 ▶); Al-Obaidi (2011 ▶); Anupama *et al.* (2012 ▶); Aytaç (2010 ▶); Hurley (2002 ▶); Saha & Koner (2004 ▶); Sreedaran *et al.* (2008 ▶).
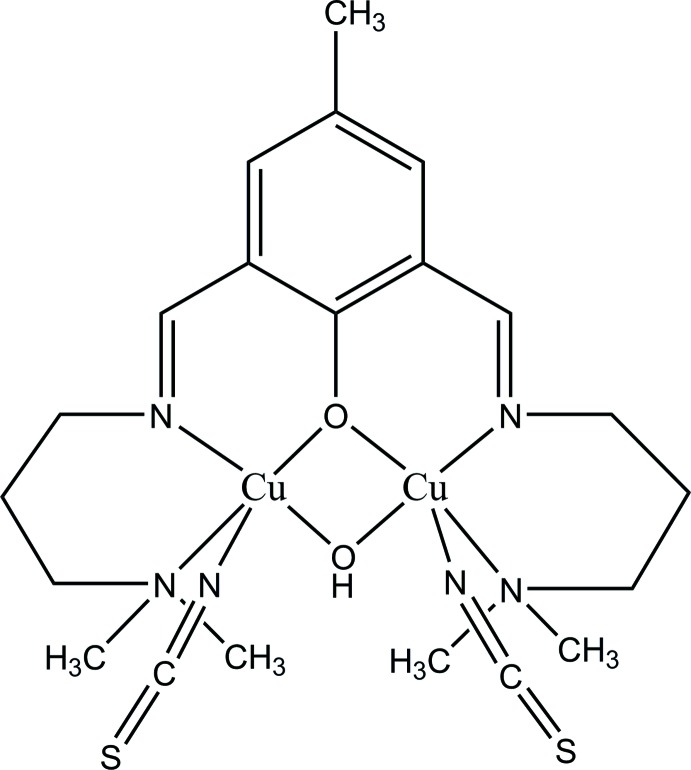


## Experimental

### 

#### Crystal data

[Cu_2_(C_19_H_31_N_4_O)(OH)(NCS)_2_]
*M* *_r_* = 591.73
Monoclinic, 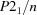

*a* = 11.9706 (5) Å
*b* = 13.7518 (7) Å
*c* = 16.9887 (8) Å
β = 109.396 (2)°
*V* = 2637.9 (2) Å^3^
*Z* = 4
Mo *K*α radiation
μ = 1.80 mm^−1^
*T* = 298 K
0.35 × 0.25 × 0.20 mm

#### Data collection

Bruker APEXII CCD area-detector diffractometer
Absorption correction: multi-scan (*SADABS*; Bruker, 2004 ▶) *T*_min_ = 0.572, *T*_max_ = 0.715
15757 measured reflections
15757 independent reflections
12691 reflections with *I* > 2σ(*I*)

#### Refinement

*R*[*F* ^2^ > 2σ(*F* ^2^)] = 0.036
*wR*(*F* ^2^) = 0.101
*S* = 1.01
15757 reflections
303 parameters
2 restraints
H-atom parameters constrained
Δρ_max_ = 0.73 e Å^−3^
Δρ_min_ = −0.53 e Å^−3^

### 

Data collection: *APEX2* (Bruker, 2004 ▶); cell refinement: *SAINT* (Bruker, 2004 ▶); data reduction: *SAINT*; program(s) used to solve structure: *SHELXS97* (Sheldrick, 2008 ▶); program(s) used to refine structure: *SHELXL97* (Sheldrick, 2008 ▶); molecular graphics: *ORTEP-3 for Windows* (Farrugia, 2012 ▶); software used to prepare material for publication: *SHELXL97*.

## Supplementary Material

Crystal structure: contains datablock(s) global, I. DOI: 10.1107/S1600536813024768/bx2448sup1.cif

Structure factors: contains datablock(s) I. DOI: 10.1107/S1600536813024768/bx2448Isup2.hkl

Additional supplementary materials:  crystallographic information; 3D view; checkCIF report

## Figures and Tables

**Fig. 1.**
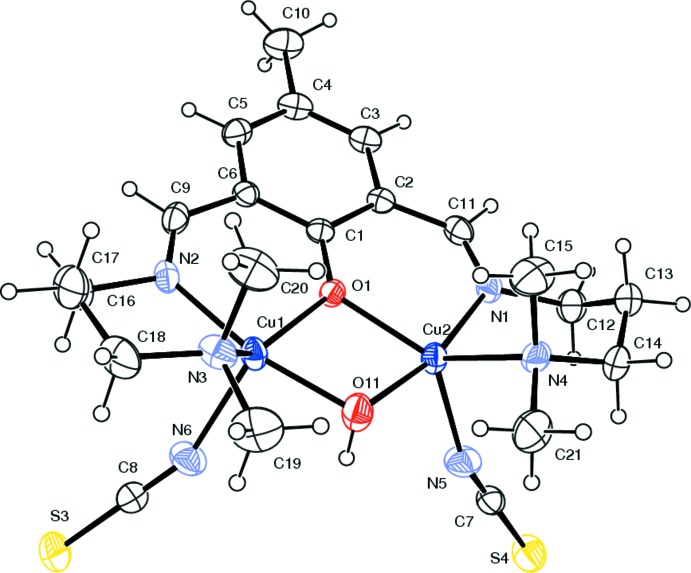
ORTEP of the molecule with atoms represented as 30% probability ellipsoids.

## supplementary crystallographic information

### 1. Comment

Binuclear copper (II) complexes are of special interest in their design and synthesis. They have distinct role to play as successful synthetic biomimetic devices capable of representing the active sites of various metalloenzymes (Adams, *et al.*, 2000). They are shown to act as possible catalysts favoring a wide variety of organic transformations in both homogeneous and heterogeneous media (Saha, *et al.*, 2004). They also serve as antifungal and antibacterial agents (Sreedaran, *et al.*, 2008; Al-Obaidi, *et al.* 2011), as DNA binding and cleaving agents (Hurley, 2002), as molecular magnetic materials and as fluorescent probes (Anupama, *et al.*, 2012). Moreover, the presence of the –C=N– groups, electronegative nitrogen, sulfur and oxygen atoms in the complex, may impart corrosion inhibition properties to the synthesized complex (Aytaç, 2010). In the title compound, C_21_H_32_Cu_2_N_6_O_2_S_2_, the molecular structure of the dinuclear cation in complex reveals two pentacoordinated cupric ions, which are bridged by the phenolate oxygen O atoms of the ligand and by an exogenous hydroxo ion. Bridging atoms occupy equatorial positions in the coordination sphere of the metals and completes its equatorial coordination plane with two N atoms of the ligand.The apical position is occupied by thiocyanate N atoms. The molecular structure is stabilized by weak O—H···N hydrogen bond interactions. The crystal structure is stablized by π-π stacking interactions involving the benzene rings [Cg1-Cg1^i^=3.764 (4)Å] ( symmetry code (i) : -x,-y,-z).The crystal studied was a non-merohedral twin with a refined BASF value of 0.2034 (20)

### 2. Experimental

A solution of 2,6-Diformyl-4-methylphenol (0.164 g, 1 mmol) in methanol was slowly added to a solution of 3-(Dimethylamino)propylamine (0.25 ml, 2.0 mmol) in 5 ml of methanol and stirred. The resulting mixture was refluxed for 10 min. To the yellow ligand solution thus obtained was added copper (II) nitrate trihydrate (0.485 g, 2 mmol) and the mixture was refluxed for another 30 min. The addition of sodium thiocyanate (0.162 g, 2 mmol) resulted in the precipitation of light green microcrystals. Single crystals suitable for X-ray diffraction were obtained by recrystallization from acetonitrile.

### 3. Refinement

All hydrogen atoms were fixed geometrically and allowed to ride on the parent carbon atoms with aromatic C—H = 0.93 Å, methylene C—H = 0.97 Å and methyl C—H = 0.96 Å. The displacement parameters were set for phenyl H atoms at *U*_iso_(H) = 1.2*U*_eq_(C) and for methylene and methyl H atoms at *U*_iso_(H) =1.5*U*_eq_(C). There was two fold twinning in the crystal. The input data was converted from HKLF 4 to HKLF 5 format for *SHELXL97* program·MERG 0 was added in the ins file and refined.

### Crystal data

**Table:**

[Cu_2_(C_19_H_31_N_4_O)(OH)(NCS)_2_]	*F*(000) = 1224
*M**_r_* = 591.73	*D*_x_ = 1.490 Mg m^−^^3^
Monoclinic, *P*2_1_/*n*	Mo *K*α radiation, λ = 0.71073 Å
Hall symbol: -P 2yn	Cell parameters from 6965 reflections
*a* = 11.9706 (5) Å	θ = 2.5–28.5°
*b* = 13.7518 (7) Å	µ = 1.80 mm^−^^1^
*c* = 16.9887 (8) Å	*T* = 298 K
β = 109.396 (2)°	Rectangular, green
*V* = 2637.9 (2) Å^3^	0.35 × 0.25 × 0.20 mm
*Z* = 4	

### Data collection

**Table:**

Bruker APEXII CCD area-detector diffractometer	15757 independent reflections
Radiation source: fine-focus sealed tube	12691 reflections with *I* > 2σ(*I*)
Graphite monochromator	*R*_int_ = 0.000
phi and ω scans	θ_max_ = 25.0°, θ_min_ = 2.3°
Absorption correction: multi-scan (TWINABS; Bruker, 2004)	*h* = −14→13
*T*_min_ = 0.572, *T*_max_ = 0.715	*k* = −16→16
15757 measured reflections	*l* = −19→20

### Refinement

**Table:**

Refinement on *F*^2^	Primary atom site location: structure-invariant direct methods
Least-squares matrix: full	Secondary atom site location: difference Fourier map
*R*[*F*^2^ > 2σ(*F*^2^)] = 0.036	Hydrogen site location: inferred from neighbouring sites
*wR*(*F*^2^) = 0.101	H-atom parameters constrained
*S* = 1.01	*w* = 1/[σ^2^(*F*_o_^2^) + (0.0461*P*)^2^ + 2.7323*P*] where *P* = (*F*_o_^2^ + 2*F*_c_^2^)/3
15757 reflections	(Δ/σ)_max_ = 0.001
303 parameters	Δρ_max_ = 0.73 e Å^−^^3^
2 restraints	Δρ_min_ = −0.53 e Å^−^^3^

### Special details

**Table:**

Geometry. All e.s.d.'s (except the e.s.d. in the dihedral angle between two l.s. planes) are estimated using the full covariance matrix. The cell e.s.d.'s are taken into account individually in the estimation of e.s.d.'s in distances, angles and torsion angles; correlations between e.s.d.'s in cell parameters are only used when they are defined by crystal symmetry. An approximate (isotropic) treatment of cell e.s.d.'s is used for estimating e.s.d.'s involving l.s. planes.
Refinement. Refinement of *F*^2^ against ALL reflections. The weighted *R*-factor *wR* and goodness of fit *S* are based on *F*^2^, conventional *R*-factors *R* are based on *F*, with *F* set to zero for negative *F*^2^. The threshold expression of *F*^2^ > σ(*F*^2^) is used only for calculating *R*-factors(gt) *etc*. and is not relevant to the choice of reflections for refinement. *R*-factors based on *F*^2^ are statistically about twice as large as those based on *F*, and *R*- factors based on ALL data will be even larger.

### Fractional atomic coordinates and isotropic or equivalent isotropic displacement parameters (Å^2^)

**Table:**

	*x*	*y*	*z*	*U*_iso_*/*U*_eq_	
C1	0.14435 (16)	0.08416 (13)	0.14686 (11)	0.0324 (4)	
C2	0.02153 (16)	0.09626 (14)	0.13278 (12)	0.0348 (5)	
C3	−0.05365 (17)	0.12155 (15)	0.05291 (12)	0.0420 (5)	
H3	−0.1340	0.1291	0.0446	0.050*	
C4	−0.01470 (18)	0.13587 (14)	−0.01390 (13)	0.0455 (5)	
C5	0.10368 (18)	0.12202 (15)	−0.00002 (13)	0.0434 (5)	
H5	0.1318	0.1300	−0.0445	0.052*	
C6	0.18437 (16)	0.09648 (14)	0.07788 (12)	0.0351 (5)	
C7	0.17786 (18)	0.19821 (16)	0.46594 (14)	0.0439 (5)	
C8	0.60543 (19)	0.19201 (15)	0.32423 (13)	0.0425 (5)	
C9	0.30672 (19)	0.08411 (16)	0.08337 (14)	0.0445 (5)	
H9	0.3224	0.0936	0.0338	0.053*	
C10	−0.0999 (2)	0.16307 (17)	−0.09907 (13)	0.0642 (7)	
H10A	−0.1784	0.1425	−0.1035	0.096*	
H10B	−0.0762	0.1317	−0.1415	0.096*	
H10C	−0.0990	0.2323	−0.1062	0.096*	
C11	−0.03213 (17)	0.08429 (16)	0.19646 (14)	0.0430 (5)	
H11	−0.1136	0.0941	0.1793	0.052*	
C12	−0.06173 (19)	0.0563 (2)	0.32495 (15)	0.0586 (7)	
H12A	−0.1417	0.0738	0.2910	0.070*	
H12B	−0.0352	0.1028	0.3703	0.070*	
C13	−0.0625 (2)	−0.0435 (2)	0.36024 (16)	0.0645 (7)	
H13A	−0.0833	−0.0900	0.3147	0.077*	
H13B	−0.1236	−0.0461	0.3860	0.077*	
C14	0.0528 (2)	−0.0738 (2)	0.42343 (14)	0.0645 (7)	
H14A	0.0759	−0.0250	0.4671	0.077*	
H14B	0.0403	−0.1342	0.4488	0.077*	
C15	0.1269 (3)	−0.16821 (18)	0.33008 (16)	0.0740 (8)	
H15A	0.1060	−0.2252	0.3546	0.111*	
H15B	0.1956	−0.1816	0.3146	0.111*	
H15C	0.0622	−0.1502	0.2813	0.111*	
C16	0.51179 (18)	0.05934 (19)	0.13281 (16)	0.0579 (7)	
H16A	0.5629	0.1092	0.1667	0.070*	
H16B	0.4998	0.0741	0.0747	0.070*	
C17	0.5721 (2)	−0.0382 (2)	0.15419 (18)	0.0704 (8)	
H17A	0.5198	−0.0876	0.1207	0.084*	
H17B	0.6430	−0.0374	0.1385	0.084*	
C18	0.60619 (18)	−0.0670 (2)	0.24424 (17)	0.0659 (8)	
H18A	0.6542	−0.1254	0.2525	0.079*	
H18B	0.6553	−0.0160	0.2778	0.079*	
C19	0.5583 (2)	−0.1110 (2)	0.36522 (16)	0.0779 (8)	
H19A	0.6109	−0.1653	0.3714	0.117*	
H19B	0.4962	−0.1278	0.3869	0.117*	
H19C	0.6016	−0.0563	0.3955	0.117*	
C20	0.4338 (2)	−0.16796 (18)	0.23131 (18)	0.0767 (8)	
H20A	0.4841	−0.2227	0.2327	0.115*	
H20B	0.3930	−0.1498	0.1744	0.115*	
H20C	0.3771	−0.1850	0.2578	0.115*	
C21	0.2588 (2)	−0.1131 (2)	0.46299 (15)	0.0758 (8)	
H21A	0.2788	−0.0594	0.5012	0.114*	
H21B	0.3241	−0.1269	0.4438	0.114*	
H21C	0.2420	−0.1693	0.4907	0.114*	
Cu1	0.393606 (19)	0.039255 (19)	0.259878 (16)	0.03997 (8)	
Cu2	0.18605 (2)	0.039242 (19)	0.329566 (15)	0.03923 (8)	
N1	0.01670 (14)	0.06201 (12)	0.27351 (11)	0.0400 (4)	
N2	0.39640 (14)	0.06200 (12)	0.14674 (11)	0.0410 (4)	
N3	0.50620 (14)	−0.08561 (13)	0.27594 (12)	0.0481 (5)	
N4	0.15297 (16)	−0.08803 (13)	0.39072 (11)	0.0458 (4)	
N5	0.20263 (17)	0.14220 (15)	0.42418 (12)	0.0598 (5)	
N6	0.52528 (17)	0.14212 (14)	0.31649 (12)	0.0591 (5)	
O1	0.21794 (10)	0.06199 (10)	0.22147 (7)	0.0381 (3)	
O11	0.35320 (12)	0.01574 (12)	0.35888 (9)	0.0573 (4)	
H11O	0.3851	0.0662	0.3891	0.086*	
S3	0.71742 (6)	0.26265 (5)	0.33508 (5)	0.0685 (2)	
S4	0.14334 (6)	0.27589 (5)	0.52589 (5)	0.0684 (2)	

### Atomic displacement parameters (Å^2^)

**Table:**

	*U*^11^	*U*^22^	*U*^33^	*U*^12^	*U*^13^	*U*^23^
C1	0.0350 (10)	0.0254 (10)	0.0345 (11)	−0.0008 (9)	0.0086 (9)	−0.0019 (8)
C2	0.0327 (10)	0.0315 (12)	0.0382 (12)	0.0025 (9)	0.0091 (9)	−0.0039 (9)
C3	0.0379 (11)	0.0365 (13)	0.0444 (13)	0.0055 (9)	0.0038 (10)	−0.0006 (10)
C4	0.0490 (13)	0.0328 (12)	0.0434 (13)	0.0036 (10)	0.0002 (10)	0.0027 (10)
C5	0.0584 (13)	0.0356 (12)	0.0363 (12)	−0.0050 (10)	0.0159 (11)	0.0030 (10)
C6	0.0384 (11)	0.0321 (12)	0.0341 (12)	−0.0051 (9)	0.0111 (9)	−0.0007 (9)
C7	0.0433 (12)	0.0416 (14)	0.0453 (14)	−0.0067 (10)	0.0128 (10)	0.0003 (10)
C8	0.0478 (12)	0.0384 (13)	0.0411 (13)	0.0020 (10)	0.0146 (10)	0.0015 (10)
C9	0.0562 (14)	0.0405 (14)	0.0452 (14)	−0.0060 (11)	0.0280 (12)	0.0002 (11)
C10	0.0714 (16)	0.0580 (15)	0.0468 (15)	0.0076 (13)	−0.0022 (12)	0.0128 (12)
C11	0.0291 (10)	0.0436 (14)	0.0554 (15)	0.0029 (9)	0.0128 (10)	−0.0073 (11)
C12	0.0412 (12)	0.085 (2)	0.0581 (16)	−0.0022 (12)	0.0274 (12)	−0.0124 (14)
C13	0.0553 (14)	0.095 (2)	0.0505 (15)	−0.0254 (14)	0.0273 (13)	−0.0086 (15)
C14	0.0776 (17)	0.0796 (19)	0.0455 (14)	−0.0293 (15)	0.0327 (13)	−0.0004 (13)
C15	0.114 (2)	0.0416 (15)	0.0677 (18)	−0.0107 (15)	0.0310 (16)	−0.0073 (13)
C16	0.0427 (13)	0.0769 (19)	0.0657 (17)	−0.0077 (12)	0.0334 (12)	−0.0049 (14)
C17	0.0498 (14)	0.085 (2)	0.087 (2)	0.0089 (14)	0.0366 (14)	−0.0185 (17)
C18	0.0357 (12)	0.0666 (18)	0.093 (2)	0.0151 (11)	0.0181 (13)	−0.0157 (15)
C19	0.0664 (16)	0.076 (2)	0.075 (2)	0.0375 (15)	0.0012 (14)	0.0123 (15)
C20	0.0779 (18)	0.0373 (15)	0.101 (2)	−0.0023 (13)	0.0113 (16)	−0.0058 (14)
C21	0.0828 (18)	0.083 (2)	0.0554 (17)	−0.0027 (16)	0.0153 (14)	0.0322 (15)
Cu1	0.02982 (13)	0.04586 (17)	0.04599 (17)	0.00438 (11)	0.01494 (12)	0.00773 (12)
Cu2	0.03448 (14)	0.04623 (17)	0.03978 (16)	0.00297 (11)	0.01607 (12)	0.00696 (12)
N1	0.0343 (9)	0.0452 (11)	0.0451 (11)	0.0004 (8)	0.0195 (8)	−0.0037 (9)
N2	0.0367 (9)	0.0420 (11)	0.0504 (11)	−0.0031 (8)	0.0228 (9)	−0.0003 (9)
N3	0.0358 (9)	0.0421 (12)	0.0599 (12)	0.0092 (8)	0.0072 (9)	−0.0043 (9)
N4	0.0599 (11)	0.0428 (11)	0.0362 (10)	−0.0100 (9)	0.0181 (9)	0.0030 (9)
N5	0.0654 (12)	0.0523 (14)	0.0605 (13)	−0.0079 (10)	0.0194 (11)	−0.0186 (11)
N6	0.0564 (12)	0.0493 (13)	0.0695 (14)	−0.0145 (10)	0.0181 (10)	−0.0130 (10)
O1	0.0266 (7)	0.0572 (9)	0.0299 (8)	0.0025 (6)	0.0084 (6)	0.0034 (6)
O11	0.0382 (8)	0.0844 (12)	0.0495 (10)	0.0098 (8)	0.0148 (7)	0.0199 (8)
S3	0.0648 (4)	0.0720 (5)	0.0766 (5)	−0.0241 (3)	0.0340 (4)	0.0000 (4)
S4	0.0654 (4)	0.0669 (5)	0.0845 (5)	−0.0065 (3)	0.0405 (4)	−0.0248 (4)

### Geometric parameters (Å, º)

**Table:**

C1—O1	1.3168 (19)	C15—H15B	0.9600
C1—C6	1.416 (3)	C15—H15C	0.9600
C1—C2	1.419 (2)	C16—N2	1.477 (2)
C2—C3	1.400 (3)	C16—C17	1.510 (3)
C2—C11	1.439 (3)	C16—H16A	0.9700
C3—C4	1.377 (3)	C16—H16B	0.9700
C3—H3	0.9300	C17—C18	1.500 (3)
C4—C5	1.370 (3)	C17—H17A	0.9700
C4—C10	1.514 (2)	C17—H17B	0.9700
C5—C6	1.400 (3)	C18—N3	1.489 (3)
C5—H5	0.9300	C18—H18A	0.9700
C6—C9	1.446 (3)	C18—H18B	0.9700
C7—N5	1.151 (3)	C19—N3	1.477 (3)
C7—S4	1.621 (2)	C19—H19A	0.9600
C8—N6	1.151 (2)	C19—H19B	0.9600
C8—S3	1.616 (2)	C19—H19C	0.9600
C9—N2	1.279 (3)	C20—N3	1.474 (3)
C9—H9	0.9300	C20—H20A	0.9600
C10—H10A	0.9600	C20—H20B	0.9600
C10—H10B	0.9600	C20—H20C	0.9600
C10—H10C	0.9600	C21—N4	1.483 (3)
C11—N1	1.281 (3)	C21—H21A	0.9600
C11—H11	0.9300	C21—H21B	0.9600
C12—N1	1.482 (2)	C21—H21C	0.9600
C12—C13	1.499 (3)	Cu1—O11	1.9255 (15)
C12—H12A	0.9700	Cu1—N2	1.9587 (17)
C12—H12B	0.9700	Cu1—O1	2.0085 (12)
C13—C14	1.499 (3)	Cu1—N6	2.0985 (19)
C13—H13A	0.9700	Cu1—N3	2.1437 (17)
C13—H13B	0.9700	Cu2—O11	1.9219 (14)
C14—N4	1.492 (3)	Cu2—N1	1.9577 (16)
C14—H14A	0.9700	Cu2—O1	2.0203 (12)
C14—H14B	0.9700	Cu2—N5	2.101 (2)
C15—N4	1.470 (3)	Cu2—N4	2.1384 (17)
C15—H15A	0.9600	O11—H11O	0.8715
			
O1—C1—C6	121.49 (17)	N3—C18—H18A	108.3
O1—C1—C2	120.93 (18)	C17—C18—H18A	108.3
C6—C1—C2	117.58 (16)	N3—C18—H18B	108.3
C3—C2—C1	119.27 (18)	C17—C18—H18B	108.3
C3—C2—C11	116.95 (17)	H18A—C18—H18B	107.4
C1—C2—C11	123.78 (18)	N3—C19—H19A	109.5
C4—C3—C2	123.35 (18)	N3—C19—H19B	109.5
C4—C3—H3	118.3	H19A—C19—H19B	109.5
C2—C3—H3	118.3	N3—C19—H19C	109.5
C5—C4—C3	116.88 (18)	H19A—C19—H19C	109.5
C5—C4—C10	121.9 (2)	H19B—C19—H19C	109.5
C3—C4—C10	121.2 (2)	N3—C20—H20A	109.5
C4—C5—C6	123.2 (2)	N3—C20—H20B	109.5
C4—C5—H5	118.4	H20A—C20—H20B	109.5
C6—C5—H5	118.4	N3—C20—H20C	109.5
C5—C6—C1	119.72 (18)	H20A—C20—H20C	109.5
C5—C6—C9	117.17 (19)	H20B—C20—H20C	109.5
C1—C6—C9	123.11 (18)	N4—C21—H21A	109.5
N5—C7—S4	179.1 (2)	N4—C21—H21B	109.5
N6—C8—S3	179.6 (2)	H21A—C21—H21B	109.5
N2—C9—C6	129.14 (19)	N4—C21—H21C	109.5
N2—C9—H9	115.4	H21A—C21—H21C	109.5
C6—C9—H9	115.4	H21B—C21—H21C	109.5
C4—C10—H10A	109.5	O11—Cu1—N2	167.19 (6)
C4—C10—H10B	109.5	O11—Cu1—O1	76.72 (5)
H10A—C10—H10B	109.5	N2—Cu1—O1	90.78 (6)
C4—C10—H10C	109.5	O11—Cu1—N6	94.66 (7)
H10A—C10—H10C	109.5	N2—Cu1—N6	95.00 (7)
H10B—C10—H10C	109.5	O1—Cu1—N6	126.15 (7)
N1—C11—C2	129.00 (18)	O11—Cu1—N3	94.82 (7)
N1—C11—H11	115.5	N2—Cu1—N3	92.17 (7)
C2—C11—H11	115.5	O1—Cu1—N3	135.42 (6)
N1—C12—C13	111.92 (19)	N6—Cu1—N3	97.86 (7)
N1—C12—H12A	109.2	O11—Cu2—N1	166.83 (7)
C13—C12—H12A	109.2	O11—Cu2—O1	76.52 (5)
N1—C12—H12B	109.2	N1—Cu2—O1	90.62 (6)
C13—C12—H12B	109.2	O11—Cu2—N5	95.13 (7)
H12A—C12—H12B	107.9	N1—Cu2—N5	94.81 (7)
C12—C13—C14	114.5 (2)	O1—Cu2—N5	126.34 (7)
C12—C13—H13A	108.6	O11—Cu2—N4	94.66 (7)
C14—C13—H13A	108.6	N1—Cu2—N4	92.19 (7)
C12—C13—H13B	108.6	O1—Cu2—N4	133.61 (6)
C14—C13—H13B	108.6	N5—Cu2—N4	99.53 (7)
H13A—C13—H13B	107.6	C11—N1—C12	116.86 (17)
N4—C14—C13	115.60 (18)	C11—N1—Cu2	125.65 (14)
N4—C14—H14A	108.4	C12—N1—Cu2	117.46 (14)
C13—C14—H14A	108.4	C9—N2—C16	116.05 (18)
N4—C14—H14B	108.4	C9—N2—Cu1	125.50 (14)
C13—C14—H14B	108.4	C16—N2—Cu1	118.35 (15)
H14A—C14—H14B	107.4	C20—N3—C19	108.8 (2)
N4—C15—H15A	109.5	C20—N3—C18	110.60 (19)
N4—C15—H15B	109.5	C19—N3—C18	107.21 (18)
H15A—C15—H15B	109.5	C20—N3—Cu1	108.21 (13)
N4—C15—H15C	109.5	C19—N3—Cu1	110.66 (14)
H15A—C15—H15C	109.5	C18—N3—Cu1	111.32 (14)
H15B—C15—H15C	109.5	C15—N4—C21	109.2 (2)
N2—C16—C17	112.44 (19)	C15—N4—C14	110.47 (19)
N2—C16—H16A	109.1	C21—N4—C14	107.06 (18)
C17—C16—H16A	109.1	C15—N4—Cu2	107.68 (14)
N2—C16—H16B	109.1	C21—N4—Cu2	110.35 (14)
C17—C16—H16B	109.1	C14—N4—Cu2	112.04 (14)
H16A—C16—H16B	107.8	C7—N5—Cu2	160.80 (18)
C18—C17—C16	115.2 (2)	C8—N6—Cu1	157.66 (19)
C18—C17—H17A	108.5	C1—O1—Cu1	129.96 (12)
C16—C17—H17A	108.5	C1—O1—Cu2	130.01 (12)
C18—C17—H17B	108.5	Cu1—O1—Cu2	100.02 (5)
C16—C17—H17B	108.5	Cu2—O11—Cu1	106.70 (6)
H17A—C17—H17B	107.5	Cu2—O11—H11O	103.5
N3—C18—C17	115.82 (18)	Cu1—O11—H11O	101.1
			
O1—C1—C2—C3	178.85 (17)	N6—Cu1—N3—C19	67.38 (16)
C6—C1—C2—C3	−1.3 (3)	O11—Cu1—N3—C18	−147.13 (15)
O1—C1—C2—C11	−1.0 (3)	N2—Cu1—N3—C18	43.61 (15)
C6—C1—C2—C11	178.84 (19)	O1—Cu1—N3—C18	136.92 (14)
C1—C2—C3—C4	−0.2 (3)	N6—Cu1—N3—C18	−51.74 (16)
C11—C2—C3—C4	179.66 (19)	C13—C14—N4—C15	−63.7 (3)
C2—C3—C4—C5	1.5 (3)	C13—C14—N4—C21	177.5 (2)
C2—C3—C4—C10	179.94 (19)	C13—C14—N4—Cu2	56.4 (2)
C3—C4—C5—C6	−1.3 (3)	O11—Cu2—N4—C15	−89.45 (16)
C10—C4—C5—C6	−179.73 (19)	N1—Cu2—N4—C15	79.30 (16)
C4—C5—C6—C1	−0.2 (3)	O1—Cu2—N4—C15	−13.65 (19)
C4—C5—C6—C9	179.91 (19)	N5—Cu2—N4—C15	174.54 (16)
O1—C1—C6—C5	−178.67 (17)	O11—Cu2—N4—C21	29.70 (16)
C2—C1—C6—C5	1.5 (3)	N1—Cu2—N4—C21	−161.55 (16)
O1—C1—C6—C9	1.2 (3)	O1—Cu2—N4—C21	105.50 (16)
C2—C1—C6—C9	−178.60 (19)	N5—Cu2—N4—C21	−66.30 (17)
C5—C6—C9—N2	179.3 (2)	O11—Cu2—N4—C14	148.89 (14)
C1—C6—C9—N2	−0.5 (4)	N1—Cu2—N4—C14	−42.37 (15)
C3—C2—C11—N1	−179.7 (2)	O1—Cu2—N4—C14	−135.31 (13)
C1—C2—C11—N1	0.1 (4)	N5—Cu2—N4—C14	52.88 (15)
N1—C12—C13—C14	66.9 (3)	S4—C7—N5—Cu2	103 (13)
C12—C13—C14—N4	−67.0 (3)	O11—Cu2—N5—C7	−179.4 (6)
N2—C16—C17—C18	−63.9 (3)	N1—Cu2—N5—C7	9.3 (6)
C16—C17—C18—N3	66.5 (3)	O1—Cu2—N5—C7	103.6 (6)
C2—C11—N1—C12	179.1 (2)	N4—Cu2—N5—C7	−83.8 (6)
C2—C11—N1—Cu2	1.3 (3)	S3—C8—N6—Cu1	117 (30)
C13—C12—N1—C11	118.7 (2)	O11—Cu1—N6—C8	165.6 (5)
C13—C12—N1—Cu2	−63.2 (2)	N2—Cu1—N6—C8	−22.9 (5)
O11—Cu2—N1—C11	−13.7 (4)	O1—Cu1—N6—C8	−117.5 (5)
O1—Cu2—N1—C11	−1.36 (18)	N3—Cu1—N6—C8	70.0 (5)
N5—Cu2—N1—C11	125.20 (19)	C6—C1—O1—Cu1	−0.5 (2)
N4—Cu2—N1—C11	−135.05 (18)	C2—C1—O1—Cu1	179.35 (13)
O11—Cu2—N1—C12	168.5 (3)	C6—C1—O1—Cu2	−179.45 (13)
O1—Cu2—N1—C12	−179.19 (15)	C2—C1—O1—Cu2	0.4 (2)
N5—Cu2—N1—C12	−52.63 (16)	O11—Cu1—O1—C1	−177.76 (16)
N4—Cu2—N1—C12	47.12 (16)	N2—Cu1—O1—C1	−0.57 (16)
C6—C9—N2—C16	−177.2 (2)	N6—Cu1—O1—C1	96.20 (16)
C6—C9—N2—Cu1	−0.9 (3)	N3—Cu1—O1—C1	−94.45 (17)
C17—C16—N2—C9	−122.6 (2)	O11—Cu1—O1—Cu2	1.45 (6)
C17—C16—N2—Cu1	60.8 (2)	N2—Cu1—O1—Cu2	178.65 (6)
O11—Cu1—N2—C9	13.6 (4)	N6—Cu1—O1—Cu2	−84.59 (9)
O1—Cu1—N2—C9	1.19 (19)	N3—Cu1—O1—Cu2	84.76 (10)
N6—Cu1—N2—C9	−125.21 (18)	O11—Cu2—O1—C1	177.76 (16)
N3—Cu1—N2—C9	136.69 (18)	N1—Cu2—O1—C1	0.62 (16)
O11—Cu1—N2—C16	−170.1 (3)	N5—Cu2—O1—C1	−95.81 (16)
O1—Cu1—N2—C16	177.44 (15)	N4—Cu2—O1—C1	94.24 (16)
N6—Cu1—N2—C16	51.04 (16)	O11—Cu2—O1—Cu1	−1.46 (6)
N3—Cu1—N2—C16	−47.05 (16)	N1—Cu2—O1—Cu1	−178.59 (7)
C17—C18—N3—C20	62.5 (3)	N5—Cu2—O1—Cu1	84.98 (9)
C17—C18—N3—C19	−179.0 (2)	N4—Cu2—O1—Cu1	−84.97 (9)
C17—C18—N3—Cu1	−57.8 (2)	N1—Cu2—O11—Cu1	14.2 (4)
O11—Cu1—N3—C20	91.13 (16)	O1—Cu2—O11—Cu1	1.56 (7)
N2—Cu1—N3—C20	−78.13 (16)	N5—Cu2—O11—Cu1	−124.62 (9)
O1—Cu1—N3—C20	15.2 (2)	N4—Cu2—O11—Cu1	135.36 (8)
N6—Cu1—N3—C20	−173.48 (16)	N2—Cu1—O11—Cu2	−14.3 (4)
O11—Cu1—N3—C19	−28.01 (16)	O1—Cu1—O11—Cu2	−1.57 (7)
N2—Cu1—N3—C19	162.73 (16)	N6—Cu1—O11—Cu2	124.51 (8)
O1—Cu1—N3—C19	−103.96 (16)	N3—Cu1—O11—Cu2	−137.17 (8)
